# Terahertz technology and its applications in head and neck diseases

**DOI:** 10.1016/j.isci.2023.107060

**Published:** 2023-06-07

**Authors:** Shenggan Shi, Shuqin Yuan, Jun Zhou, Peidu Jiang

**Affiliations:** 1 Department of Pharmacy, Personalized Drug Therapy Key Laboratory of Sichuan Province, Sichuan Provincial People’s Hospital, University of Electronic Science and Technology of China, Chengdu, China; 2 School of Electronic Science and Engineering, University of Electronic Science and Technology of China, Chengdu, China

**Keywords:** Disease, Radiation biology

## Abstract

The terahertz (THz) radiation refers to electromagnetic waves between infrared and millimeter waves. THz technology has shown a significant potential for medical diagnosis and biomedical applications over the past three decades. Therefore, exploring the biological effects of THz waves has become an important new field in life sciences. Specifically, THz radiation has been proved to be able to diagnose and treat several head and neck diseases. In this review, we primarily discuss the biological characteristics of THz waves and clinical applications of THz technology, focusing on the research progress of THz technology in head and neck diseases (brain cancer, hypopharyngeal cancer, oral diseases, thyroid nodules, Alzheimer’s disease, eyes diseases, and otitis). The future application perspectives of THz technologies in head and neck diseases are also highlighted and proposed.

## Graphical abstract

**Figure:**
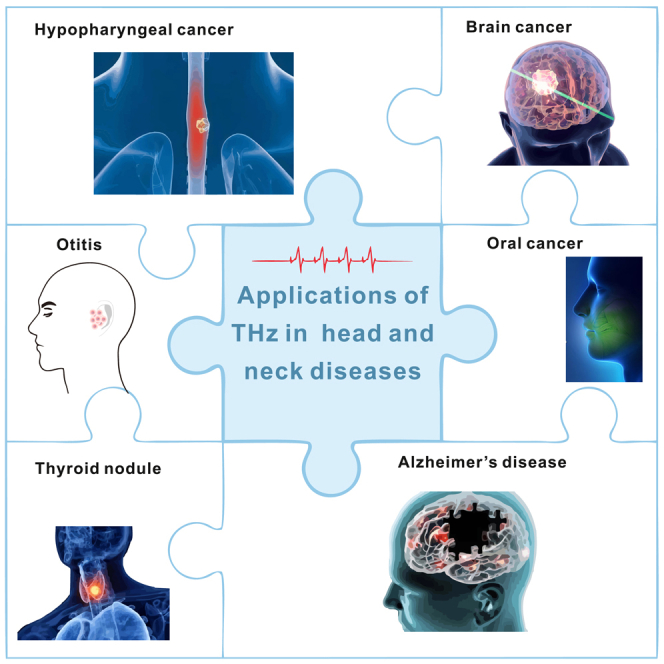

## Introduction

**Figure 1:**
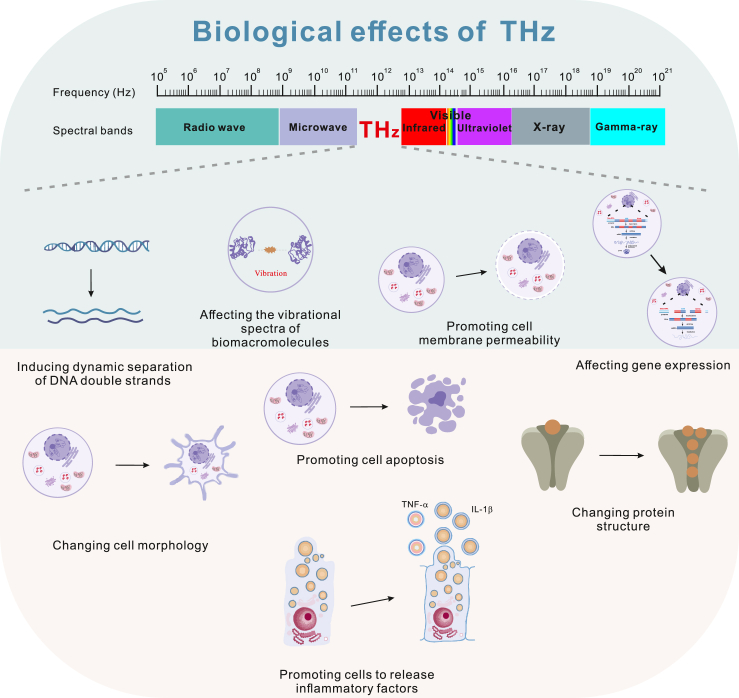
Biological effects of THz

Terahertz (THz) radiation, wavelength range from 3,000 to 30 μm, is an electromagnetic wave between the infrared and microwave with a frequency region from 0.1 to 10 THz (1 THz = 10^12^ Hz).^1^ In the past, the terahertz band was referred to as the “terahertz gap” due to the lack of effective generators and detection techniques for a long time. However, in recent decades, the rapid development of ultrafast laser technology and semiconductor materials science has provided stable and reliable laser sources for pulse generation, promoting the application of THz radiation in spectroscopy and imaging technology. At present, extensive studies have greatly broadened our understanding of head and neck diseases, such as brain cancer, hypopharyngeal cancer, oral diseases, etc. Numerous tools have been developed for therapeutic and diagnostic purposes. In such studies, interdisciplinary and cutting-edge approaches from physics and biophysics play a vital important role. Due to its unique biological characteristics (Figure 1), THz is widely applied in the treatment and diagnosis of head and neck diseases.

Head and neck diseases are a general term for a group of diseases that occur in the head and neck, including head and neck cancer (HNC), neurodegenerative diseases, eye diseases, otitis, etc. Among them, HNC is the main type of head and neck diseases. HNC is a category of cancers usually located in the squamous cells of nose, mouth, throat, and neck. HNC was the ninth most common cancer worldwide in 2020, accounting for 8.2% (19,292,789 new cases) of all cancers and over 4.3% (9,958,133 deaths) of all cancer deaths worldwide.^2^ HNC were often diagnosed in older patients associated with heavy use of tobacco and alcohol,^3^ and late diagnosis severely affected the patient’s survival rates. Fortunately, the prevalence of HNC was slowly declining globally, partly due to decreased use of tobacco.^4^

**Figure 2:**
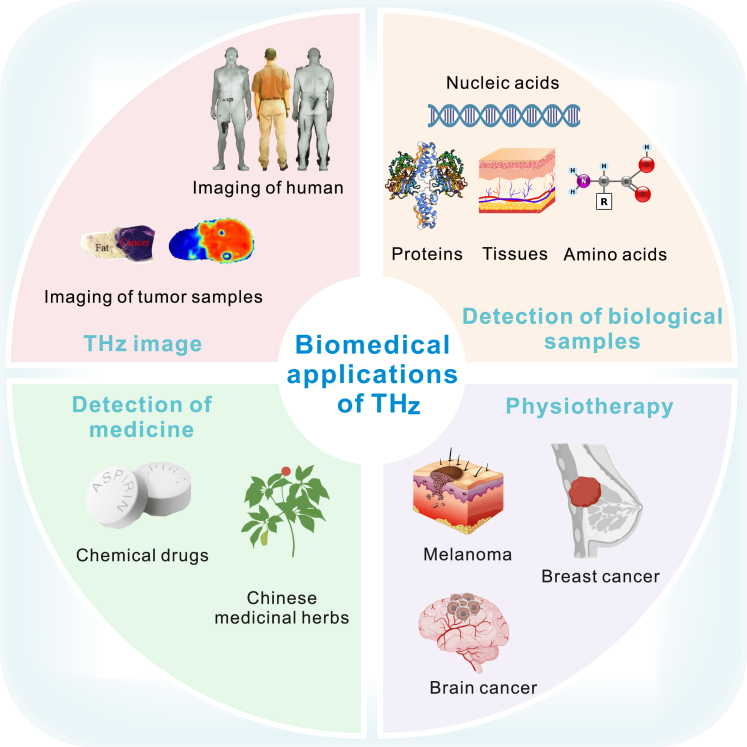
Biomedical applications of THz (partially modified from Figure 4 of ref.^6^)

**Figure 3:**
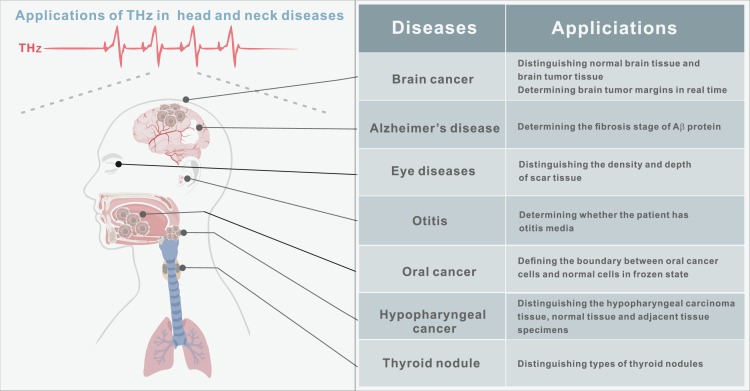
Applications of THz in head and neck diseases

Over the past few decades, a variety of techniques have been developed to generate and detect THz radiation.^5^ Currently, THz coherent measurement techniques allow for direct measurement of the amplitude and phase of oscillating electromagnetic fields, accurately measuring the refractive index and absorption coefficient of samples, surpassing traditional optical methods by a significant margin. Subsequently, due to their high sensitivity to biomolecules and water content, THz-based methods hold tremendous potential in biomedical research and diagnostics (Figure 2). Finally, the properties of THz imaging and spectroscopy (e.g., non-ionizing nature of THz radiation, making it safe for human exposure at low power) make these THz detection techniques promising candidates for head and neck disease research (Figure 3). This article provides a concise overview of the biological characteristics of THz radiation, with a particular focus on the research progress regarding the biological effects of THz waves in head and neck diseases. Furthermore, it discusses the applications of THz technology in head and neck diseases. Finally, it highlights some unresolved issues and emphasizes new directions for future research.

## Biological characteristics of THz technology

Due to the unique optical properties of THz, several biological characteristics have been widely utilized for the detection and identification of bio-organic molecules or tissues. The biological properties of THz radiation in the detection and identification are broadly classified into the following categories.

### Non-ionizing and non-invasive

The photon energy of electromagnetic wave with frequency of 1 THz is only about 4.14 meV, which is about 1/10^6^ of X-ray photon energy.^7^^,^^8^ Compared with X-rays, THz photon radiation energy is several energy levels lower and has non-ionizing characteristics, which does not cause ionization hazard effects like X-rays. This is an advantage for biologic samples because the low energy of THz photon radiation is not sufficient to ionize biomolecules from disturbed tissues,^9^ nor does it produce significant thermal effects or determine electron leap spectra, and therefore can be used more safely for *in vivo* real-time diagnosis and periodic screening and monitoring. However, it is crucial for us to note that although THz radiation is non-ionizing radiation, it can become a biohazard when used improperly. Therefore, using of THz technology for diagnostic applications should be performed at a controlled radiation power density and exposure time.^10^

### Unique THz characteristic spectra

THz waves have unique spectral features for identifying molecules in the THz range by evaluating their specific spectral features.^7^ The energy of molecular rotation and vibrational transition determines the specific spectrum of intermolecular interactions, and the spectrum of low-frequency inter- and intra-molecular motions (hydrogen bond, molecular vibrations, molecular rotations, van der Waals forces, etc.) of biomolecules falls in the THz band, and different biomolecules can be identified by analyzing and identifying the unique THz signature spectra generated by these activities.^11^ The distribution of hydrogen bonds can also be revealed by THz imaging.^12^ In addition, the THz signal is influenced by the specific overexpressed of some proteins during tumorigenesis or progression.^13^

### Phase-sensitive to polar compounds

Polar molecules exhibit higher absorption of THz waves and thus the phase sensitivity of water and body fluid levels shows stronger absorption and better contrast than X-rays. He et al. reported that water exhibited strong absorption at THz frequencies (α = 200 cm^−1^ at 1.0 THz).^14^ As the water concentration increases, the ability of THz radiation to pass through the medium diminishes. Hence, THz radiation has different responses to cell and tissue pairs with different water contents, which has led to widespread interest in THz for the detection and identification of different kinds of tumors.^12^ However, the methods of distinguishing tumor and normal tissue based on the difference water contents is a double-edged sword. Because on the one hand, it can accurately distinguish tumor from normal tissue by using different water contents of cancer tissue and normal tissue; on the other hand, high absorption of water may affect the accuracy of the results.^15^ In addition, since bacteria have different water contents in different states of survival (lag phase, logarithmic phase, stationary phase, or stationary phase), THz can also be used to detect the survival state of bacteria based on the changes of hydration levels.^7^

### Ability to penetrate non-polar molecules

Many materials are opaque in the visible and near-infrared regions, which greatly limits their further analysis. Interestingly, these materials go on to be visible in the THz band,^7^ which is one of the most attractive features of THz radiation. Due to the phase sensitivity of THz to moisture, the materials will show sharp contrast due to the difference in water content.^16^ For example, THz waves can penetrate non-polar molecules such as plastic and paper, which can be transmitted through these materials to achieve the non-destructive detection of packaging content without unpacking.

### High temporal-spatial resolution and signal-to-noise ratio capabilities

The typical pulse width of THz waves is on the sub-picosecond scale, allowing for sub-picosecond and femtosecond time-resolved transient spectroscopy studies,^17^ which can provide time-resolved analysis of the collective vibrational modes of biomolecules, as well as spatially resolved near-field spectroscopy at specific micron scales.^18^ At the same time, due to the high frequency and short wavelength, THz waves have high signal-to-noise ratio in time domain spectrum.

### Non-thermal effects of THz radiation

Organisms can absorb part of the THz energy and convert it into heat energy. Therefore, thermal effects are the characteristic of THz radiation. But THz radiation can also produce non-thermal effects on biological systems under the condition of controlling temperature. At present, *in vivo* studies of the non-thermal biological effects of THz radiation have focused on rat and mouse models, with the main observed effects focusing on histopathological changes in the skin, wound healing, and changes in blood sample indices (Table 1).^19^^,^^20^^,^^21^^,^^22^^,^^23^^,^^24^

**Table 1:** *In vivo* studies of non-thermal effects of THz radiation

Biological models	Biological effects	Possible mechanisms	References
Human	Induced DNA double bond breakage causes DNA damage in bare skin tissue	Increased expression level of phosphorylated histone H2AX (γH2AX)	Titova et al.^19^
Rat	Promoting platelet clumping in the blood	Selective absorption of metabolites (NO, O_2_, CO_2_, CO) at 0.15 THz	Kirichuk et al.^20^
	Depression level increased after exposure	Unclear	
	Changing in lipid peroxidation intermediates, antioxidant indicators in blood	NO selective absorption to 0.15 THz	
	Improvement of coagulation, fibrinolysis, and nitrite indexes in blood after exposure to restraint stress	Unclear	Kirichuk et al.^21^
Mice	Induction of acute inflammation of skin tissue	Unclear	Hwang et al.^22^
	1. Differential expression of genes related to wound response was observed in skin tissues 2. Prolonged wound healing, slowed epithelialization and increased scar area were found	Wound response activation, TGF-β signaling pathway activation	Kim et al.^23^
Drosophila	Gene expression changes in Drosophila	Unclear	Vi et al.^24^

### Unmarkedness and biological safety of THz radiation

THz waves hold a unique position in the electromagnetic spectrum, bridging the fields of electronics and photonics, which endow them with distinct properties such as their unmarked nature. The unmarked nature of THz waves enables direct examination of molecules in their natural state, facilitating the comprehension of molecular interactions without the requirement of artificial probes or labels. As a result, the data obtained is more likely to reflect the innate behavior of these molecules. This is highly beneficial for understanding the relationship between structure, activity, disease research, and the discovery of safe and effective drugs. Additionally, the favorable biosafety of THz is a significant factor driving its widespread application in the field of biomedicine. For instance, Bogomazova et al. found that exposure to 2.3 THz had no significant impact on the morphology and mitosis index of human embryonic stem cells, did not induce the formation of γH2AX foci and structural chromosome aberrations, and did not result in significant changes in gene expression levels. These finding suggested that THz radiation did not cause DNA damage or gene expression changes in human embryonic stem cells.^25^ Furthermore, Liu et al. conducted a one-week exposure of the eyes of eight rabbits to 0.3 THz radiation and assessed the safety of THz radiation on the eye at the tissue, cellular, structural, and functional levels. The results indicated that the cornea and lens remained transparent after THz irradiation, and the activity of corneal stromal cells and endothelium was unaffected. There were no significant changes observed in mRNA expression levels associated with DNA loss and cell growth.^26^

Although effective THz radiation generation and detection methods were initially lacking, such as specific and sensitive sources and detectors,^27^^,^^28^ which limited our understanding of the nature of electromagnetic radiation band. However, with the rapid development and application of new technologies and materials, especially ultrafast laser technology, THz-TDS and THz imaging, scientists have made great progress in the study of the generation mechanism, detection methods, and application techniques of THz radiation.^29^ Table 2^6^^,^^30^^,^^31^^,^^32^^,^^33^^,^^34^^,^^35^^,^^36^^,^^37^^,^^38^^,^^39^^,^^40^^,^^41^^,^^42^^,^^43^^,^^44^^,^^45^^,^^46^^,^^47^^,^^48^^,^^49^^,^^50^^,^^51^^,^^52^^,^^53^^,^^54^^,^^55^^,^^56^^,^^57^^,^^58^^,^^59^^,^^60^ outlines the potential of THz technology at the biomolecular and biological tissue levels, in molecular spectroscopy, pharmaceutical engineering, medical imaging, and oncology.

**Table 2:** Relevant biomedical applications of THz technology and related conclusions

Techniques	Detected Compounds		Results	References
THz time-domain spectroscopy (THz-TDS)	Amino acids and peptides	Glycine, alanine and their peptides	The vibrational band of poly-glycine was found at a frequency of 1.37 THz	Yamamoto et al.^30^
	Nucleic acids	DNA	Recording the far-infrared (0.5–4.0 THz) dielectric function of the four nucleobases and corresponding nucleosides forming the building blocks of deoxyribose nucleic acid (DNA)	Fischer et al.^31^
		Sequences of oligonucleotides	The four oligonucleotides(HQ-411, HQ-418, HQ-419, and HQ-420)exhibited distinguishably different absorption behaviors over a frequency range of 0.2–2.6 THz	Tang et al.^32^
		RNA	The single-stranded RNA polymers poly-A and poly-C showed significant differences in both absorption and refractive index in the frequency range of 0.1–3 THz	Fischer et al.^33^
	Proteins	TRPM8 protein	It is the first study of the glycosylated state of proteins from the perspective of molecular structure and cellular functional expression using THz spectroscopy	Mernea et al.^34^
		Immunoglobin (IgG) protein	Determination of the effects of two antibodies on the dielectric properties of a polar liquid from 0.1 to 1.3 THz	Sun et al.^35^
		Six kinds of protein rn21, rn22, rn28, n42, n43 and n53	A label-free method to acquire the quantitative distribution of different kinds of proteins using THz-TDS was presented	Han et al.^36^
	Lipids	Myelin	It demonstrated differences in the optical properties of myelin-deficient and normal brains in the THz band	Zou et al.^37^
	Tissues	Fresh porcine muscle, fresh porcine adipose tissue and skin	Measurement of the optical properties from 0.1 to 1.6 THz for muscle, adipose tissue, and skin	Wilmink et al.^38^
		Human dehydrated normal and cancerous gastric tissues	THz characteristic spectra of gastric cancer tissues were observed at 0.2–0.5 THz and 1–1.5 THz	Hou et al.^39^
		Human brain tissues from Alzheimer’s disease patients	It showed differences between healthy and diseased tissues	Png et al.^40^
		Brain tissues from AD mice	It was the first time to detect optical molecular torsional modes due to tryptophan difference between AD and normal mouse models using THz technique	Shi et al.^41^
		Human shin	THz features of the palm, ventral (inner) and dorsal (outer) forearm in the 0.1–2 THz spectral region were collected	Echchgadda et al.^42^
	Pharmaceuticals	Aspirin and aspirin precursors	The reduction of the spreading of the drug compounds of aspirin and its precursors was found by THz-TDS	Laman et al.^43^
		Tablet release properties	THz-TDS technology allowed fast (1 s) direct measurement of porosity of different types of tablets	Bawuah et al.^44^
THz pulsed imaging (TPI) and THz imaging	Tissues	Laboratory rats	TPI allowed non-invasive and accurate differentiation of tissue types	Huang et al.^45^
		Basal cell carcinoma and melanoma	Tumors exhibited high absorption areas in both pulsed THz imaging (0.2–0.5 THz integrated)	Berry et al.^46^
	Proteins	Immunoglobulin G (IgG) and ferritin molecules	It was the first demonstration of THz near-field imaging of a single IgG molecule	Yang et al.^47^
	Pharmaceuticals	Comparation of the efficacy of different TDD methods	THz imaging technique for quantitative evaluation of different transdermal application methods was feasible and potential	Wang et al.^48^
		Tablet and coating integrity and performance	THz radiation can extract information on the internal coating configuration of a table	Fitzgerald et al.^49^
	Cancers	Skin cancer	THz pulse imaging can distinguish basal cell carcinoma from normal tissue	Woodward et al.^50^ Pickwell et al.^51^ Wallace et al.^52^
		Breast cancer	TPI can distinguish breast cancerous tissue from normal tissue	Vohra et al.^53^ Bowman et al.^6^
		Oral cancer	THz imaging can distinguish well between cancerous areas and surrounding tissues in the oral region	Sim et al.^54^^,^^55^
		Brain cancer	THz imaging can determine the boundaries of malignant regions using animal brain tumor models	Oh et al.^56^
THz-based metallic mesh (electromagnetic surface waves in the THz region)	Nucleic acids	Single-stranded and double-stranded of DNA	A metallic mesh-based THz method clearly revealed the difference in optical properties between single- and double-stranded DNA molecules on the basis of refractive index	Hasebe et al.^57^
THz photo-mixing spectrometer	Nucleic acids	DNA	A strong signal around 0.72 THz and a weak signal around 0.4 THz were detected in the aqueous DNA solution	Zhang et al.^58^
THz freeze out spectroscopy (TFOS)	Proteins	Protein-ligand binding	TFOS can be used to identify protein-ligand binding in solution	Chen et al.^59^
All-metal THz metamaterial biosensor	Proteins	Bovine serum albumin	THz biosensor based on all-metal metamaterials can detect proteins well	Wang et al.^60^

## THz-related technologies and clinical applications

### Frozen THz imaging

Limited by the strong absorption of THz by water molecules, THz radiation cannot penetrate into wet biological tissues deeply. For instance, THz radiation penetrates only a few hundred microns of human skin.^61^ To overcome this problem, several groups have presented to use of freezing techniques to enhance the transmission depth of THz radiation into wet tissue,^55^^,^^62^^,^^63^^,^^64^^,^^65^ as the absorption coefficient of ice is lower than liquid water, which can excellently increase the THz radiation penetrated depth.^61^ Sim and co-workers found that due to lack of liquid water, frozen tissue had better contrast, and there were significant structural differences between frozen (−20°C) oral malignant melanoma and normal oral mucosa.^55^ Another research also showed that THz detected frozen tumor tissue with a depth of 1.3 mm, while in unfrozen oral tissues tumor tissues were not found at the same location due to THz radiation inability to reach that depth.^54^ In addition, freezing technology can also be used to differentiate malignant and benign regions of tissue that are difficult to distinguish under fresh conditions (sufficient water content). Li et al. reported that THz accurately distinguished tumors and normal tissues and sensitively detected spectral changes in adjacent tissues.^66^

### THz endoscopy and otoscopy

Currently, THz detection is limited to detect tumors in superficial tissues or close to the epithelial layer (e.g., breast cancer, melanoma), while tumors under the skin or mucosa need to be detected by other techniques or under certain conditions (e.g., with the help of penetration-enhancing agents). However, for the oral cavity, digestive organs, respiratory organs, middle ear, and other visceral lesions, diseases can only be explored through laparoscopy, bronchoscopy, otoscope, and other endoscopies. Fortunately, after great efforts, THz technology has been combined with these endoscopes and applied to the diagnosis of diseases in these tissues and organs. Ji et al. fabricated and characterized a small THz endoscopic system using a photoconductive generator and detector with a cross-section of (2 × 4 mm) × 6 mm,^67^ which was small enough to be easily inserted into the human body. They then used this small handheld THz endoscope to measure the THz optical coefficients of the human mouth and tongue.^68^ This group also developed and fabricated a THz otoscope to assist doctors diagnose otitis media (OM) by measuring changes in the water content of the tympanic membrane caused by tissues hydration.^69^

### Penetration-enhancing agents and materials

It is worth emphasizing that water has strong interference to THz. Although freezing technique can improve the penetration depth of THz, it has some limitations in clinical applications as it can occasionally damage tissues during the freezing process. Therefore, researchers have proposed the use of special materials or penetration-enhancing agents (PEAs) to increase the contrast and permeability of THz. Ahi and Kashanian et al. used gold nanoparticles to increase the contrast of THz spectroscope,^70^ and others used metamaterials with high refractive index to enhance the contrast.^71^ Musina et al. revealed the interaction between the enhanced penetration depth and diffusion rate of THz in hyperosmotic agents and noted that as glycerol, PG, and PEG with relative molecular masses of 200 and 400 absorbed less THz waves, this greatly enhanced the penetration depth of THz waves to tissues [68]. Another group also found that glycerol as a PEA significantly increased the transmission depth of THz radiation in fresh wet tissues.^72^

### THz-CT

Computed tomography (CT) detection is commonly used to diagnose the internal properties of materials. However, due to series of limitations of CT detection (e.g., radiation, and X-ray will damage the material structure), the application range of CT detection is greatly limited. Therefore, combination of a safe THz time-domain spectroscopy (THz-TDS) system and CT imaging methods to achieve low-cost nondestructive detection of complex objects has become an important development direction for THz technology. THz-tomography uses a THz-TDS system as a carrier to achieve spatial information reconstruction technology in CT scans through simultaneous rotation and translation.^73^ Fosodeder et al. proposed a new method for THz CT image reconstruction and quantitative evaluation of reconstructed 3D printed plastic profiles, revealing the potential of this method for nondestructive testing of plastic profiles. This also provided a new approach for nondestructive testing of medical materials.^74^

### THz-based sensors and microfluidics

THz microfluidics is a technique for analyzing biological particles by THz-related techniques in an extremely small volume of liquid.^75^ In addition to imaging for *in vivo* detection of diseases, THz microfluidics can facilitate the development of *in vitro* diagnostic medical devices (IVDs). THz IVDs enable rapid diagnosis of minute amounts of specimens (e.g., nucleic acids, proteins, or various metabolites) at the molecular level. For instance, Geng and co-workers prepared two THz metamaterial biosensors with integrated microfluidics and found that the liver cancer biomarkers alpha-fetoprotein (AFP) and glutaminyl transferase isozyme II (GGT-II) showed resonance shifts of about 19 GHz (5 mu/ml) and 14.2 GHz (0.02524 μg/mL).^76^ Based on THz attenuated total reflection (ATR) microfluidic cells and THz-TDS system, Tang et al. reported a novel label-free fast THz spectroscopy technique, which detected different DNA molecules (e.g., normal hemoglobin gene, sickle cell anemia gene, JAK2 gene wild type, and JAK2 V617F gene mutation from sickle cell anemia and thrombocytopenia).^77^

## The application of THz imaging and spectroscopy in head and neck diseases

With the continuous advancement of medical technology, people’s understanding of diseases has evolved significantly. Unfortunately, most cancers remain incurable as they are often diagnosed at advanced stages. Therefore, early diagnosis of these malignant diseases is crucial for prevention, improving survival rates, and reducing the risks associated with complex surgeries and late-stage cancer treatments. Currently, clinical methods used for the diagnosis and staging of head and neck diseases include physical examinations, laryngoscopy, X-ray computer tomography (CT), magnetic resonance imaging (MRI), positron emission tomography (PET) scan, tissue biopsy, etc. However, each screening method has its unique advantages and disadvantages (e.g., cost, specificity, invasiveness, radiation exposure) (Table 3).

**Table 3:** Comparison of imaging diagnosis methods

Techniques	safety	Limitations	References
X-ray computed tomography	Less radiation exposure	Low sensitivity to several tisues	Wang et al.^78^
MR	Non-invasive	High cost; time-consuming	Stucht et al.^79^
PET	Minimal radiation exposure	High cost; poor inflammation specificity	Moses et al.^80^
THz	Non-invasive and non-ionizing under controlled radiation power density and exposure time	As discussed in Part 6	Yang et al.^7^

As mentioned in the previous section, THz technology offers unique biological advantages and has widespread applications in disease diagnosis and treatment. In addition to the biological advantages mentioned above, THz technology also has the following advantages: THz waves have a wavelength that falls between microwaves and infrared, providing strong complementarity to other electromagnetic waves. Compared to near-infrared imaging techniques, THz technology can effectively avoid interference from particle scattering effects. Additionally, the broad frequency characteristics of THz waves facilitate the analysis of the spectral properties of substances over a wide range.

There is substantial evidence indicating that THz can be used as a useful tool for early identification of head and neck diseases, as THz radiation detection methods provide more accurate detection of malignant tissue’s boundaries and depth of infiltration.^56^^,^^81^

### Brain cancer

As one of the most important organs of the human body, the brain regulates the basic and important physiological activities of our daily life (breathing, memory, and speech, etc.). Therefore, precise surgery is absolutely necessary for brain cancer patients, as it can both reduce damage to normal brain tissue and eliminate the malignant tumor to the maximum extent. Fortunately, since the brain is rich in sphingolipids, the brain is an organ with a high-lipid content, which makes it possible to easily distinguish the distribution areas of brain cancer by THz imaging. Because brain tumors contain a higher concentration of proteins, which have a higher absorption of THz radiation, and a lower concentration of lipids, THz imaging has a higher contrast to distinguish brain cancer.

Oh et al. successfully distinguished brain tumor tissue from normal brain tissue in an animal brain tumor model using reflectance-based THz imaging. Tumor boundaries determined from THz images were consistent with those determined from conventional images.^56^

A study of 26 human glioma specimens embedded in gelatin revealed a statistically significant difference in THz optical constants between normal brain tissue and grade I-IV gliomas, suggested that THz pulse spectroscopy was an effective tool for differentiating normal tissue from grade I-IV gliomas.^82^

Ji et al. overcame the critical limitation of detection in glioma surgery by using THz reflectance imaging (TRI) to successfully identify the border between glioma and normal brain tissue. By comparing with H&E staining, optical coherence tomography (OCT), fluorescence imaging, and other imaging approaches, they found that TRI could well distinguish tumor regions.^83^

### Hypopharyngeal cancer

Li et al. investigated the role of THz technology in determining the pathological margins of hypopharyngeal carcinoma by constructing the xenograft model of hypopharyngeal carcinoma in BALB/c nude mice. The results of spectral analysis showed that the THz absorption coefficient of the tumor tissue of hypopharyngeal carcinoma nude mice transplantation tumor was significantly higher than that of the normal tissue, while the THz absorption coefficient of the adjacent tissue was between the normal tissue and the tumor tissue, indicating that the THz technique distinguished the hypopharyngeal carcinoma tissue, normal tissue, and adjacent tissue specimens.^66^

### Oral diseases

Oral diseases include oral precancer and cancer, dental caries. Among them, oral cancer is one of the most common malignant tumors of the head and neck and is a general term for malignant tumors occurring in the oral cavity, including gum cancer, tongue cancer, floor of mouth cancer, and oropharyngeal cancer. Therefore, it is necessary to accurately distinguish the tumor boundary for oral cancer surgery to minimize the damage for patients. Sim et al. detected the samples of seven patients with oral cancer by freezing technology and THz endoscopy. The results showed that THz detection well defined the boundary between cancer cells and normal cells in frozen state. As mentioned in 3.1, the background effect of water on THz imaging can be well suppressed under freezing condition.^54^ Dental caries, also known as tooth decay or worm teeth, is a kind of oral disease that dental tissue is decayed, gradually destroyed and disintegrated, and forms a caries hole. THz detection technology detected the degree and depth of caries lesions through the change of refractive index in enamel.^27^^,^^84^ Compared with the naked eye detection, THz detection technology can detect smaller changes.

### Thyroid nodule

Thyroid nodules are solitary nodules inside the thyroid. Thyroid tumor nodules, cystic nodules, and inflammatory nodules can be clearly distinguished by radiological examination. Konnikova et al. studied the plasma of patients with malignant thyroid nodules and healthy people by THz-TDS and robotic-learning methods. The results showed that the plasma samples of patients with malignant thyroid nodules had higher THz absorption than those of healthy people.^85^

### Alzheimer’s disease

Alzheimer’s disease has become a growing concern. The best way to treat Alzheimer’s disease is to detect the disease as early as possible so that the disease can be treated earlier and more effectively. In recent years, researchers have reported the use of THz-related technology for the early detection of Alzheimer’s disease. Shi and co-workers compared the THz absorptivity and refractive index of brain tissue from AD model mice and normal control wild-type mice. The results showed that three absorption peaks closer to free tryptophan were observed in AD tissues but not in normal tissues, and therefore, the THz tryptophan absorption pattern can be used as a biomarker profile for AD in the brain.^41^ Notably, this study was the first to use THz technology to detect optical molecular torsion patterns caused by tryptophan differences between AD models and normal mice. Heo et al. determined the fibrosis stage of Aβ protein by observing the change of near-field THz conductivity.^86^ The results showed that the conductivity of Aβ monomer was very high, while the conductivity decreased in Aβ oligomers and dropped to an insulating state in Aβ fibrils.

### Eye diseases

Ke et al. studied the corneal scar tissue isolated from rabbit by using pulsed THz broadband spectrum and imaging.^87^ The authors evaluated four corneal samples and confirmed that THz-TDS was an effective tool to distinguish the density and depth of scar tissue. The distribution of the density of scar tissue, the change of optical properties of scar site and the change of scar depth were obtained by THz-TDS and image reconstruction.^87^ Ke et al. utilized visible light of specific wavelengths to induce elastic changes in the rabbit cornea and employed THz technology to observe structural variations in the cornea. By capturing THz signals within the cornea and measuring the refractive index changes caused by corneal strain, they calculated the elastic Young’s modulus of the cornea. In the future, this research can contribute to understanding the mechanisms underlying corneal deformation under intraocular pressure conditions.^88^ Furthermore, Ke et al. investigated corneas with different water contents using THz spectroscopy and discovered that it can non-invasively characterize corneal sublayers, providing information about various corneal sublayers.^89^^,^^90^

### Otitis

Otitis media (OM) refers to the general term for various inflammations occurring in the middle ear. At present, the diagnosis method is to determine the presence of otitis media by observing the presence of pus through optical otoscope or surgical puncture of tympanic membrane. Ji et al. designed and fabricated a THz otoscope that determined if you have otitis media by observing changes in the water content of the eardrum.^69^^,^^91^

## Limitations of the study

Currently, the application of THz-related technologies in the medical field is experiencing rapid development. However, as an emerging technology, THz has certain limitations: *Low resolution:* the resolution of THz imaging typically ranges from 20 μm to 200 μm, which has not yet reached the level of conventional optical microscopy. *Sensitivity to temperature variations and water absorption:* THz waves are highly sensitive to temperature changes, and water molecules strongly absorb THz radiation, which can potentially affect the accuracy of THz measurements. *High cost:* the relatively high cost of THz systems hampers its widespread applications in different hospitals and healthcare centers. *Bio-safety:* the biosafety of high energy THz needs further investigation. *Inability to differentiate specific stages of tumors:* THz technology lacks the capability to distinguish specific stages of tumors. *Repeatability and accuracy:* the repeatability and accuracy of the experimental results in different research groups were quite different.

## Future directions

Nevertheless, the field of THz technology and theory continues to mature and evolve. To address the limitations mentioned above, we can explore solutions in the following areas.

(1) For the problem of low resolution: improvements can be made in addressing the issue of low resolution by employing sample pre-processing techniques, adjusting THz optical paths, and optimizing imaging methods. In the case of water absorption affecting the measurement, sample pre-processing techniques such as paraffin embedding or freezing, along with the use of THz penetration enhancers like glycerol, can be helpful in mitigating the effects of water absorption on measurements. For soft biological samples, fixation with porous materials or agarose prior to measurement can reduce deformation. Additionally, microfluidic and nanofluidic devices can be utilized to minimize water absorption losses in THz measurements. As for adjustment of THz optical path and imaging, the problem of low resolution can be improved by changing the optical path of THz to improve the signal-to-noise ratio, and the resolution can also be optimized by computer algorithms after THz imaging.
(2) For the problem of temperature sensitivity: in experiments involving THz non-thermal effects, constructing a temperature-controlled platform can help maintain a constant external temperature, minimizing the impact of external temperature on experimental results.
(3) For the problem of high cost: with the ongoing development in the fields of physics and materials science, coupled with the exploration of novel terahertz sources such as spintronic terahertz sources, it is believed that low-cost and efficient terahertz sources can be realized, addressing the issue of high cost.
(4) For the issue of biosafety: it is a prerequisite for future use of THz radiation. It is necessary to establish a set of quantifiable safety criteria to evaluate the biosafety of THz technology, regarding the differences in application scenarios, irradiation sites and irradiation times. It is also necessary to consider the long-term consequences of chronic THz exposure on human beings from the biological tissue perspective and comprehensively explore the mechanism of THz waves effects on various structural levels of organisms.
(5) For the problem of distinguishing the specific stages of tumors: as tumor diseases are in a continuous development process, if accurate identification of cancer stages is required, terahertz technology can be used to detect the content and types of biomarkers in cancer tissues at different stages. In the future, with the continuous development of biotechnology and medical technology, THz imaging combined with THz spectral fingerprints of biomarkers simultaneously achieve qualitative identification of cancer regions and quantitative analysis of cancer development stages.
(6) For problems with poor experimental reproducibility and significant differences in accuracy: it is first necessary to strengthen research collaboration among different research groups, scientifically standardize THz radiation parameters, and improve the reproducibility and comparability of experimental results. Additionally, accelerated acquisition speed, improved signal-to-noise ratio, and higher power equipment should be used to enhance the accuracy and repeatability of analysis results. Finally, we can improve our understanding of the interaction mechanisms between samples and THz radiation by employing theoretical models, such as computational models based on molecular dynamics simulations. Commercial software can also be utilized for numerical simulation of terahertz spectra.

## Conclusion

The unique photoelectric properties of THz waves make it a frontier research field. Since there is a very complex interaction between THz waves and biomacromolecules, exploring the biological effects and mechanism of THz radiation has become a hot research topic in life science. THz radiation technology has become a potentially useful tool in medical field due to the advancement of THz wave emission source and detection technology.

With the perfect of theory and maturity of industrial application, the reduction of equipment cost and large-scale popularization, THz radiation has great potential applications in disease diagnosis and treatment, especially in non-destructive, label-free, real-time clinical analysis. Nowadays, the development of science and technology has entered the information age. Whether THz technology can be more closely related to the latest advanced technologies such as big data, cloud computing, and blockchain is the direction that researchers need to work together. For example, in the context of precision medicine, more accurate medical imaging technology is needed to guide individualized and accurate treatment for patients with different characteristics. In the future, THz technology can be integrated with advanced computer technologies such as big data analysis and artificial intelligence to better serve patients.
